# High Concentrations of Very Long Chain Leaf Wax Alkanes of Thrips Susceptible Pepper Accessions (*Capsicum* spp)

**DOI:** 10.1007/s10886-020-01226-x

**Published:** 2020-10-22

**Authors:** Mirka Macel, Isabella G. S. Visschers, Janny L. Peters, Nicole M. van Dam, Rob M. de Graaf

**Affiliations:** 1 Molecular Interaction Ecology, Institute of Water and Wetland Research (IWWR), Radboud University, P.O. Box 9010, 6500 GL Nijmegen, The Netherlands; 2 Institute of Biology, Plant Ecology and Phytochemistry, Leiden University, P.O. Box 9505, 2300 RA Leiden, The Netherlands; 3 Plant Systems Physiology, Institute of Water and Wetland Research (IWWR), Radboud University, P. O. Box 9010, 6500 GL Nijmegen, The Netherlands; 4 German Centre for Integrative Biodiversity Research (iDiv) Halle-Jena-Leipzig, Deutscher Platz 5e, 04103 Leipzig, Germany; 5 Institute of Biodiversity, Friedrich Schiller University Jena, Dornburger-Str. 159, 07743 Jena, Germany; 6 Microbiology, Institute of Water and Wetland Research (IWWR), Radboud University, P.O. Box 9010, 6500 GL Nijmegen, The Netherlands

**Keywords:** Capsicum, Wax metabolites, Alkanes, Triterpenoid, Thrips, Resistance, Plant defense

## Abstract

The cuticular wax layer can be important for plant resistance to insects. Thrips *(Frankliniella occidentalis*) damage was assessed on 11 pepper accessions of *Capsicum annuum* and *C. chinense* in leaf disc and whole plant assays. Thrips damage differed among the accessions. We analyzed the composition of leaf cuticular waxes of these accessions by GC-MS. The leaf wax composition was different between the two *Capsicum* species. In *C. annuum, 1-*octacosanol (C_28_ alcohol) was the most abundant component, whereas in *C. chinense 1-*triacotanol (C_30_ alcohol) was the prominent. Thrips susceptible accessions had significantly higher concentrations of C_25_-C_29_ *n*-alkanes and *iso*-alkanes compared to relatively resistant pepper accessions. The triterpenoids α- and ß-amyrin tended to be more abundant in resistant accessions. Our study suggests a role for very long chain wax alkanes in thrips susceptibility of pepper.

**Electronic supplementary material:**

The online version of this article (10.1007/s10886-020-01226-x) contains supplementary material, which is available to authorized users.

## Introduction

The cuticular wax layer of the leaf is a first barrier for an herbivorous insect to tackle after landing on the leaf surface. Plant cuticular waxes can consist of very long chain (> 20C) alkanes, ketones, alcohols, fatty acids and triterpenoids (Eigenbrode and Espelie 1995). The primary function of this wax layer is to protect the plant against desiccation, solar radiation and pathogens (Jenks et al. 1995; Mariani and Wolters-Arts 2000). The wax layer can be a physical barrier for insects to attach to or penetrate the leaf surface (Eigenbrode and Espelie 1995). The triterpenoids and other metabolites in the wax layer can be feeding or oviposition deterrents for herbivorous insects (Eigenbrode and Pillai 1998). For example, amyrins (triterpenoids) reduced feeding by diamandback moth larvae on cabbage (Eigenbrode and Pillai 1998). On the other hand, some wax metabolites can also be used for host plant recognition and as feeding stimulants by various insects such as sawflies and flea beetles (Braccini et al. 2015; Mitra et al. 2017; Müller and Hilker 2001; Udayagiri and Mason 1997). Relatively high amounts of leaf wax have been associated with higher susceptibility against thrips in onions and leak (Damon et al. 2014). This means that the chemical composition of the wax layer can have positive and negative effects on herbivore resistance.

Thrips (Thysanoptera) are a major agricultural pest world-wide. In greenhouses in Europe, western flower thrips (*Frankliniella occidentalis*) is a generalist pest on many crops. Thrips are sucking piercing insects that cause deformations and stunted growth, and cause damage by spreading viruses (Steenbergen et al. 2018). Recent bans on pesticides and increasing resistance to the insecticides that are still used call for identifying natural sources of resistance in crops. Plant metabolites linked to thrips resistance are amongst others alkaloids (Liu et al. 2019), chlorogenic acid (Leiss et al. 2009), tocopherols (Maharijaya et al. 2012) and diterpene glycosides (Macel et al. 2019; Maharijaya et al. 2018). However, in most studies the entire leaf was analyzed and not specifically the wax compounds. Most of the above-mentioned compounds are not present in the epicuticular wax layer, although very small traces of alkaloids have been found in the wax layer of leaves of *Senecio jacobaea* (Vrieling and Derridj 2003). This means that the contribution of wax layer chemistry, which commonly consist of apolar components, to thrips resistance are relatively unknown.

Peppers (*Capsicum* spp., Solanaceae) are grown world-wide and damage by several thrips species causes great economic losses (Shipp et al. 1998, Visschers 2020). Commercially grown peppers generally belong to the species *Capsicum annuum* and *C. chinense*. Because of their economic interest, there has been quite some research on the wax layers of the pepper fruits in relation to food quality and food preservation (de Rijke et al. 2015). Major components of the fruit wax layer were C_29_ and C_31_ *n*-alkanes and C_24_ fatty acid (Bauer et al. 2005; de Rijke et al. 2015; Parsons et al. 2013). Both plant ontogeny and leaf age play a role in cuticular wax metabolite composition (Busta et al. 2017). In addition, abiotic and biotic factors influence plant waxes (Shepherd and Griffiths 2006). For example, bacterial root endophytes can influence the concentrations of wax metabolites in the leaves and fruits (Silva et al. 2014). How leaf wax metabolites relate to thrips resistance in peppers has not been studied yet.

Here, we analyzed the leaf cuticular wax layer composition of previously identified thrips resistant and susceptible *Capsicum annuum* and *C. chinense* accessions, using Gas Chromatograph coupled to Mass Spectrometry (GC-MS). We grew the 11 selected accessions in the greenhouse and analyzed the cuticular wax metabolites of young leaves from flowering plants. We related differences in wax chemical composition to the outcome of thrips (*F. occidentalis*) leaf disc and whole plant preference and performance assays. An earlier study that analyzed entire leaves of nine Capsicum accessions of four species (*C. annuum, C. chinense, C. frutescens, C. baccatum*) showed that thrips susceptible accessions had relatively high concentrations of C_25_-C_28_ *n*-alkanes (Maharaijya et al. 2012). Because alkanes are the major constituents of the wax layer, we hypothesized that the concentration of *n*-alkanes in the epicuticular wax would be higher in thrips susceptible *Capsicum* accessions. Triterpenoids (phytosterols) are known to be involved in constitutive and herbivore induced plant defenses (Eigenbrode and Pillai 1998; Zhang et al. 2018). Therefore, we hypothesized that triterpenoid concentrations in the wax layer would be higher in thrips resistant *Capsicum* accessions.

## Materials and Methods

### Leaf Wax Metabolites GC-MS Analyses

#### Plant Material

Seeds of all accessions were obtained from the Center for Genetic Resources Netherlands (Table S1) and selected based on prior knowledge on insect resistance. The seeds were multiplied in the greenhouses of the Radboud University Nijmegen. Seeds were germinated on glass beads and seedlings transferred to 1.5 L pots filled with commercial potting soil 1–2 weeks after germination. The pots were placed on tables in a greenhouse, inside an insect-free net cage (Rovero 0.30 mm gauze, 7.50 m x 3 m x 2.75 m) at 16 h photoperiod and minimum temperatures set to 20 °C/17 °C (day/night). Natural light was supplemented with Greenpower lights (400V/1000W, Phillips, Amsterdam, the Netherlands) when below 200Watt m-2. Predatory mites, *Amblyseius swirskii* (Koppert Biological Systems, Berkel en Rodenrijs, the Netherlands), were released in the greenhouse to control for accidental thrips infection. Plants were transferred to 3L pots when they were three months old and provided with nutrients once a week.

### Thrips Resistance Assessments

#### Leaf Disc No-choice Assay

These experiments were previously published as part of Visschers et al. 2019a. The purpose of no-choice assays was to determine thrips resistance of the pepper accessions *per se* and not thrips preference. In brief, three plants of the selected accessions were grown in the greenhouse. After four months, when all plants were flowering, one standardized leaf of each plant was collected. Leaf discs (1.5 cm diameter) were punched from the leaves. One leaf disc of each accession was put in a small petri dish with five L1/L2 *F. occidentalis* larvae (reared on green beans) and left to feed for 48hrs. Leaf damage was assessed using imaging software (Visschers et al. 2018a, b).

#### Leaf Disc Choice Assay

These experiments were previously published as part of Macel et al. 2019. In brief, the same accessions were used in a leaf disc choice assay. Ten plants of each accessions were grown in the greenhouse and leaf discs were taken from a standardized set of leaves of four months old flowering plants. One disc of each accession was placed in a petridish, which contained in total 11 discs (one of each accession, in randomized order for each petri dish). Twenty-two L1/L2 *F. occidentalis* larvae were added to the petri dish and left to feed for 48hrs. Leaf damage was assessed in the same manner as the no-choice leaf disc assay.

#### Whole Plant Assay

Seeds of the eleven accessions were sown in potting trays for germination and transferred to 1L pots with potting soil. One to three plants per accession were grown individually in closed nylon mesh bags (1 m length x 0.5 m diameter) in the greenhouse. Temperatures were set to 24/24 °C and light was supplemented when below 400 W/m² using 10.000 lux Son-T lamps. When the plants were four weeks old they were infested with 50 adult *F. occidentalis* females. Five weeks after thrips inoculation, the damage on the plants was scored on a scale from one to nine (1 = severe damage, 9 = no damage). The number of larvae and adult thrips on the plants was counted by washing the entire plant with ethanol and filtering out the thrips.

#### Wax Layer Metabolite Extraction

Methods adapted from Haslam and Kunst (2013). Two leaves, the second pair from the top (similar to the leaves used in the thrips leaf disc experiments), were collected from each plant (*n* = 2–7 plants per accession) when they were four months old and flowering. Wax metabolites were extracted by dipping the leaves for 30 seconds in 10 ml chloroform with 10 µl internal standard (tetracosane (Sigma-Aldrich) 1 ug/µl chloroform solution) using a glass vial. Chloroform was evaporated under a stream of nitrogen gas (5.0) and the wax residue resuspended in 200 µl chloroform and transferred to a glass 1.5 ml vial. After evaporation of the chloroform, 10 µl dried pyridine and 10 µl BSFTA (*N,O*-Bis(trimethylsilyl)trifluoroacetamide) (Merck) were added and the vials were sealed with phenolic polytetrafluoroethylene (PFTE) lined caps. Samples were incubated at 80° C for 1 hour. Samples were allowed to cool off and evaporate, after which the samples were resuspended in 40 µl chloroform and transferred to glass 1.5 ml vials with 200 µl inserts.

#### Leaf Area

Directly after dipping in chloroform, the leaves were placed flat on a transparent sheet with a centimeter and photographed. Leaf surface area was calculated using the magic wand tool in Adobe Photoshop CC 2018.

#### Gas Chromatography – Mass Spectrometry (GC-MS) Settings

GC-MS analyses were performed on an Agilent 7890A GC (Agilent Technologies, Santa Clara, CA, USA) equipped with a HP-5MS column (30 m x 0.25 mm x 0.25 µm) and an autosampler (7693A), injector temperature 250°, interface temperature 250 °C. The GC was connected to a JEOL AccuTOF-GCv JMS-100 mass spectrometer (JEOL Ltd., Akishima, Tokyo, Japan). For the analysis, 2 µl of each sample was injected onto the GC column using a split ratio of 10:1 and the following temperature program: 50 °C for 2 min., ramp 40 °C/min. to 200 °C, hold 1 min., ramp 3 °C/min. to 320 °C, hold 13.25 min. using a helium (5.0 ) column flow of 1.0 ml/min Electron Impact Spectra were acquired at 10 Hz (spectra per second) mass range 35–650.

#### Data Processing

GC-MS peaks were manually integrated using MassCenter (JEOL Ltd., Akishima, Tokyo, Japan). Peaks after 16 minutes were selected, which included all wax metabolites and not the cutin metabolites (Fernandez-Moreno et al. 2016). Peaks were identified based on MS spectra (NIST library) and reference standards (C_21_-C_40_ *n*-alkanes, *1-*octacosanol, α-amyrin (Sigma-Aldrich), ß-sitosterol, stigmasterol (LGC)). *Iso*-alkanes were identified by their [M-43]^+^ peak (Fernandez-Moreno et al. 2016). Five unidentified peaks were present in all samples in similar proportions and accounted for < 5% of the total wax content (data not shown). Each peak area was corrected for total leaf area and the internal standard to obtain the concentration of metabolites in µg/dm^2^ leaf area.

### Statistical Analyses

Statistical analyses were performed in R version 3.5.1 (R core team 2008). Differences in damage among the accessions in the thrips leaf disc choice assay were tested with a Friedman-ANOVA for dependent samples. The differences between accessions in the no-choice assays (whole plant and leaf disc) were analyzed with ANOVA, except for the damage classes in the whole plant assay which were analyzed with a non-parametric Kruskal-Wallis test. Accessions were set as fixed factors in these models. Leaf wax metabolites were first analyzed with Principal Component Analyses (PCA) for overall differences among the two *Capsicum* species. Difference in concentrations of individual metabolites between resistant and susceptible accessions within each species (*C. annuum* or *C. chinense*) were analyzed with non-parametric Mann-Whitney U-tests. *P*-values were corrected for multiple comparisons with FDR correction, and significance levels set at *P* < 0.014. Differences in total wax content between resistant and susceptible accessions were tested with ANOVA with resistance as fixed factor, total wax content data were log transformed to meet the assumption of normal distribution and homoscedasticity.

## Results

### Thrips Resistance Assessment

The classification of relative thrips resistance or susceptibility of the accessions was based on the leaf disc choice assays (Table 1, Macel et al. 2019). This resistance classification was compared with resistance in whole plant thrips performance assays and in no-choice leaf discs feeding damage assays (Visschers et al. 2019a) (Table 1). All three thrips tests showed that accession 43 is consistently susceptible, whereas accession 63 and 23 were the most resistant *C. annuum* accessions in all assays (Table 1). Thrips resistance of the other accessions was variable among the different assays. Accession 52 received the most damage in the leaf disc choice assay and was also one of the most susceptible accessions in the whole plant assay. Accession 19 was relatively resistant in the leaf disc assays, but less so in the whole plant test where it harbored a high number of thrips adults and larvae. Accession 34 showed a reverse pattern, being relatively susceptible in the leaf disc choice assay, but more resistant in the no-choice whole plant test. The *C. chinense* accessions were all relatively resistant in the no-choice whole plant assay, but in the no-choice leaf disc assay accession 13 and 70 were more susceptible (Table 1).

**Table 1:** Thrips (*Frankliniella occidentalis*) preference (damage % in choice assay) and performance (numbers of larvae and adults) on *Capsicum annuum* and *Capsicum chinense* accessions in different tests (data of the leaf disc choice test from Macel et al. 2019, data of the leaf disc no-choice test from Visschers et al. 2019a)

			Means *C. annuum* accessions							Means *C. chinense* accessions				
Scale	Test	Trait	14-S	34-S	43-S	52-S	19-R	23-R	63-R	38-S	13-R	41-R	70-R	*P-*value
Leaf disc	Choice	Damage (%)	13.9	13.9	18.3	20.6	4.6	5.8	1.9	9.8	4.5	2.8	4.1	<0.001
	No-choice	Damage (mm^2^)	9.4	6.5	19.7	3.1	2.8	1.2	1	7.3	23.2	5.0	26.1	<0.001
Whole plant	No-choice	Damage (level 1- 9)	5.5	5.7	2.2	3.0	4.2	5.6	5.7	7.8	7.5	8.0	8.2	<0.001
		Larvae	73	75	441	204	283	30	74	7	23	16	5	< 0.001
		Adults	92	30	122	172	44	40	47	9	19	7	7	< 0.001

RU accessions numbers are given, R indicates an accession classified as resistant, S susceptible as determined in Macel et al. 2019. Damage levels at the whole plant tests range from 1 (severe damage) to 9 (no damage). *P*-values of Friedman-ANOVA (leaf disc choice test) and ANOVA (no-choice tests: damage mm^2^, larvae and adults) or Kruskal-Wallis (no-choice test: damage level) for differences among accessions

### Leaf Wax Metabolites

**Fig. 1:**
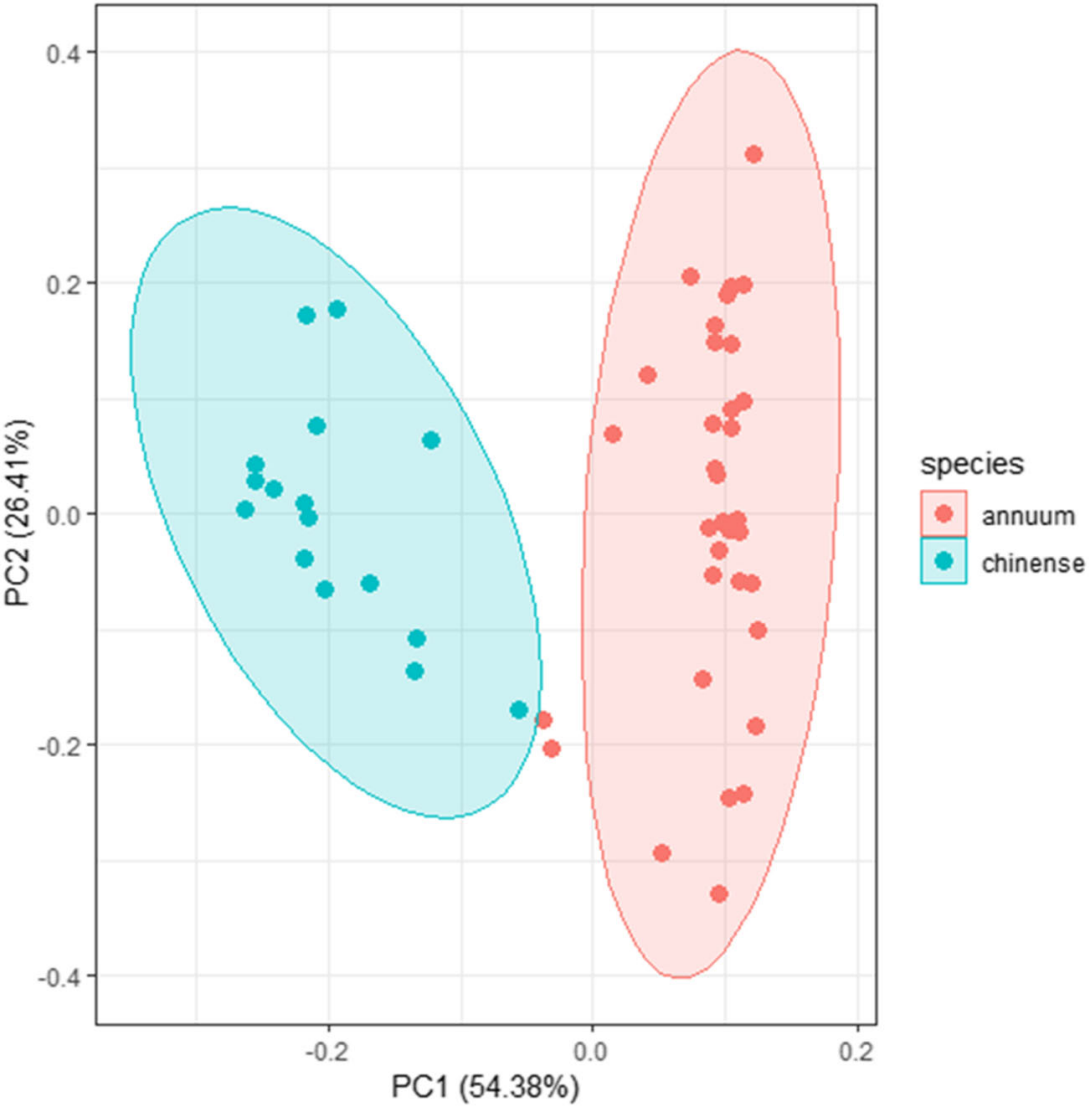
Principal component analysis plot of the relative abundance of 35 epicuticular leaf wax metabolites of *Capsicum annuum* (red dots) and *Capsicum chinense* (blue dots). The dots represent individual plant samples

The GC-MS cuticular wax analyses of leaves of the 11 accessions of the two *Capsicum* species yielded 35 metabolites (Table 2). These metabolites belonged to the classes of *n*-alkanes, branched *iso*-alkanes, long chain alcohols, and triterpenoids. We also detected a tropane alkaloid, identified by a NIST library match of 965 and accurate mass as tropacocaine, in most of the *C. annuum* accessions (Table 2, Table S2, Figure S1). The PCA plot of all data showed that leaves of *C. annuum* and *C. chinense* differed in wax metabolite composition (Fig. 1). Most metabolites were present in all samples, but *1-*octacosanol (C_28_ alcohol) was the most abundant wax metabolite of *C. annuum*, while *1-*triacotanol (C_30_ alcohol) was the most abundant wax metabolite of *C. chinense* (Table 2).

**Table 2:** Mean concentrations of cuticular wax compounds in µg/dm^2^ (± SE) of leaves of thrips resistant and susceptible *Capsicum* accessions

				*Capsicum annuum*			*Capsicum chinense*		
Compound Class	Compound	Formula	Rt	Resistant (*n* = 18)	Susceptible (*n* = 16)	*P*	Resistant (*n*=11)	Susceptible (*n*=5)	*P*
tropane alkaloids	Tropacocaine	C_15_H_19_NO_2_	9.73	0.21 (0.09)	0.03 (0.02)	0.18	0	0	n.a.
*n*-alkanes	Pentacosane	C_25_H_52_	18.14	0.03 (0.02)	0.24 (0.07)	**0.001**	0	0	n.a.
	Hexacosane	C_26_H_54_	20.19	0.02 (0.01)	0.44 (0.09)	**< 0.001**	0.01 (0.01)	0	0.83
	Heptacosane	C_27_H_56_	22.33	4.35 (0.91)	23.43 (2.87)	**< 0.001**	3.40 (1.30)	0.58 (0.44)	0.052
	Octacosane	C_28_H_58_	24.42	0.44 (0.12)	1.54 (0.22)	**< 0.001**	0.40 (0.15)	0.05 (0.05)	0.11
	Nonacosane	C_29_H_60_	26.53	4.40 (0.53)	10.41 (0.83)	**< 0.001**	4.32 (1.15)	2.52 (1.55)	0.052
	Triacotane	C_30_H_62_	28.58	0.46 (0.06)	0.64 (0.09)	0.43	0.24 (0.05)	0.57 (0.42)	0.74
	Hentriacotane	C_31_H_64_	30.65	13.91 (1.28)	17.46 (2.46)	0.89	12.33 (3.00)	11.36 (4.65)	0.66
	Dotriacotane	C_32_H_66_	32.62	2.83 (0.36)	2.27 (0.55)	0.13	1.84 (0.46)	1.29 (0.38)	0.69
	Tritriacotane	C_33_H_68_	34.61	23.23 (3.51)	28.06 (5.71)	0.61	12.20 (3.33)	11.76 (3.67)	0.91
	Tetratriacotane	C_34_H_70_	36.47	1.05 (0.22)	1.23 (0.33)	0.47	0.12 (0.04)	0.29 (0.08)	0.090
	Pentatriacotane	C_35_H_72_	38.35	5.49 (1.43)	11.32 (2.52)	0.26	0.98 (0.44)	2.85 (0.98)	*0.027*
	Heptatriacotane	C_37_H_76_	41.92	0.46 (0.08)	0.18 (0.04)	*0.027*	0.06 (0.05)	0.32 (0.11)	**0.005**
*iso-*alkanes	*iso*-nonacosane	C_29_H_60_	25.72	0.08 (0.04)	2.14 (0.23)	**< 0.001**	0	0	n.a.
	*iso*-hentriacotane	C_31_H_64_	29.86	0.86 (0.19)	2.41 (0.30)	**0.001**	0.57 (0.19)	0.29 (0.18)	0.11
	*iso*-tritiacotane	C_33_H_68_	33.86	2.71 (0.36)	5.56 (0.87)	**0.006**	3.81 (1.03)	1.92 (0.84)	0.22
	*iso*-pentatriacotane	C_35_H_72_	37.64	0.46 (0.11)	1.70 (0.29)	**0.001**	0.25 (0.14)	0.24 (0.12)	0.58
	*iso*-hexatriacotane	C_36_H_74_	39.70	0.03 (0.01)	0.26 (0.06)	**0.002**	0.18 (0.10)	0.19 (0.13)	0.99
	*iso*-heptiatriacotane	C_37_H_76_	41.25	0.01 (0.01)	0.18 (0.04)	**0.001**	0	0.02 (0.02)	0.58
other branched alkanes long-chain alcohols	branched alkane	C_32_H_66_	32.11	0.38 (0.13)	0.57 (0.11)	0.17	0.26 (0.08)	0.27 (0.12)	0.83
	branched alkane		36.77	0.05 (0.03)	0.19 (0.07)	0.15	0.04 (0.02)	0.08 (0.03)	0.22
	1-hexacosanol	C_26_H_54_O	27.58	0.41 (0.16)	1.25 (0.35)	*0.015*	0	0	n.a.
	1-heptacosanol	C_27_H_56_O	29.62	0.06 (0.03)	0.33 (0.11)	0.14	0	0	n.a.
	1-octacosanol	C_28_H_58_O	31.70	50.88 (11.7)	83.25 (20.7)	0.73	14.51 (5.65)	30.47 (9.23)	0.18
	1-nonacosanol	C_29_H_60_O	33.57	2.96 (0.67)	6.16 (1.55)	0.20	3.70 (1.47)	4.44 (1.15)	0.22
	1-triacotanol	C_30_H_62_O	35.53	22.64 (4.91)	40.91 (7.79)	0.21	86.61 (30.2)	82.19 (28.8)	0.74
	1-henatriacotanol	C_31_H_64_O	37.31	0.16 (0.06)	0.39 (0.15)	0.65	1.65 (0.57)	0.74 (0.17)	0.83
	1-dotriacontanol	C_32_H_66_O	39.15	0.36 (0.14)	1.36 (0.82)	0.47	12.90 (4.21)	9.06 (3.01)	0.91
Esters triterpenoids	alkyl ester		32.92	0.09 (0.06)	0.96 (0.33)	**0.009**	0	0.03 (0.02)	0.22
	Stigmasterol	C_29_H_48_O	34.26	0.09 (0.04)	0.12 (0.05)	0.63	0.12 (0.04)	0.29 (0.08)	0.38
	ß-sitosterol	C_29_H_50_O	35.33	5.98 (1.26)	10.56 (2.64)	0.29	2.44 (0.93)	0.48 (0.29)	0.069
	Triterpenoid		35.69	1.60 (0.51)	3.64 (0.91)	*0.089*	0	0	n.a.
	ß*-*amyrin	C_30_H_50_O	36.0	4.90 (1.07)	3.13 (0.55)	0.19	3.44 (1.11)	0.20 (0.09)	**< 0.001**
	α-amyrin	C_30_H_50_O	36.19	3.05 (0.73)	1.68 (0.60)	*0.047*	1.86 (0.90)	1.61 (0.39)	0.38
	Triterpenoid		37.88	0.44 (0.27)	0.22 (0.09)	0.39	0	0.03 (0.03)	0.58
Total	total wax			155.49 (22.86)	282.86 (41.36)	0.040	168.43 (53.70)	164.37 (52.39)	0.66

*C. annuum*, three resistant and four susceptible accessions; *C. chinense*, three resistant and one susceptible accession. *n* = total number of individual plants used for analyses. Rt = retention time. *P*-values of Mann-Whitney U-tests of differences between resistant and susceptible plants within each *Capsicum* species, with correction for multiple comparisons (FDR, *P* < 0.014 significant, indicated in bold). Marginally significant *P* values (*P* < 0.05) are in italics

Wax metabolites varied among the different accessions (Table S2). Within *C. annuum*, thrips susceptible accessions had on average significantly higher levels of C_25_-C_29_ *n*-alkanes as well as of some *iso*-alkanes and an unknown alkyl ester, compared to resistance accessions (Table 2). For *C. chinense*, the susceptible accessions had significantly higher levels of C_37_ *n*-alkane. Resistant *C. chinense* had higher levels of the phytosterol β-amyrin (Table 2). Our analyses did not reveal any metabolites that had significantly higher concentrations in thrips resistant accessions of *C. annuum*, although there is a trend for higher levels of α-amyrin in the wax layer of resistant accessions (*P* = 0.047, Table 2). Total wax content tended to be higher in susceptible accessions of *C. annuum* compared to resistant accessions (*P* = 0.04, Table 2).

## Discussion

Our analyses of the cuticular wax metabolites of the leaves of *Capsicum* accessions showed that accessions that were relatively susceptible to western flower thrips had higher concentrations of cuticular *n*-alkanes and branched *iso*-alkanes than accessions that were more resistant. Triterpenoid amyrins tended to be higher in more resistant pepper accessions. The thrips assays could consistently identify the most and least resistant *Capsicum* accessions, even though the assays varied a little with regards to the exact ranking of the accessions.

Our study suggests that susceptibility to western flower thrips in pepper plants correlates with high concentrations of wax alkanes. Onion thrips (*Thrips tabaci*) also preferred onion accessions with high concentrations of epicuticular *n*-alkanes and total wax content (Damon et al. 2014). Partly, the effect of wax chemical composition depends on the insect and the plant species (Eigenbrode and Espelie 1995). Some insects can use the plant wax metabolites, and specifically the alkanes, as oviposition stimulants (e.g. Spencer 1996, Müller and Hilker 2001, Mitra et al. 2017). The reasons why thrips or other insects prefer plants with more alkanes are thus far unknown. Possibly, the alkanes of the plant cuticular wax layer act as feeding stimulant to thrips. The epicuticula of insects consists of compounds very similar to the plant waxes, and insects may acquire these cuticular hydrocarbons through their food (Silverman and Liang 2000). Strikingly, the epicuticula of *F. occidentalis* consists of C_25_ - C_29_ *n*-alkanes (Gołebiowski et al. 2007), and exactly these same *n*-alkanes were more abundant in cuticular waxes of thrips susceptible *Capsicum* accessions. Whether thrips, like ants, acquire alkanes through their food is still unknown (Silverman and Liang 2000). It is also possible that other defensive traits that are correlated with wax alkanes determine thrips feeding damage. A third possibility could be that low amounts of alkanes increases the permeability of the cuticle, and more easily exposes plant defense metabolites to the leaf surface (Bessire et al. 2007).

*C. annuum* and *C. chinense* accessions had distinct leaf wax compositions. Wax metabolite profiles are known to be plant species specific (Mariani and Wolters-Arts 2000), which may be why insects have evolved to use wax components as reliable oviposition cue to select their host plant species. Wax composition also varies with plant age and plant organ (Lee and Suh 2015) and can change upon herbivory (Zhao et al. 2020). In our study of waxes of pepper leaves, C_27_, C_31_ and C_33_ *n*-alkanes were the most abundant alkanes. The major alkanes in pepper fruit wax were C_29_ and C_31_ *n*-alkanes (Bauer et al. 2005; de Rijke et al. 2015; Parson et al. 2013), which shows that the wax composition may differ among organs. Genes involved in biosynthesis of cuticular waxes have been identified in *Arabidopsis* and other models species such as *Hordeum* (Lee and Suh 2015). The pepper genome has been sequenced (Kim et al. 2014), but the effort to unravel the genes involved in cuticular wax biosynthesis in pepper is not as advanced as in *Arabidopsis* or tomato. So far, mainly candidate genes involved in cuticle development have been identified (Popovsky-Sarid et al. 2017). Next to genetic factors, abiotic conditions such as drought stress can also influence cuticular wax composition (Shepherd and Griffiths 2006). In addition, microbes and insects, for example feeding by the Hessian fly, can alter plant leaf wax profiles (Aragón et al. 2017; Kosma et al. 2010; Silva et al. 2014). Leaf cuticular wax composition is thus both genetically and environmentally determined. Furthermore, microbes that live in or on the plant surface can also produce specific metabolites (Schmidt et al. 2018). Endophytic fungi are known to produce tropane alkaloids (Naik et al. 2018). It is possible that the tropane alkaloid we detected in low amounts in the pepper leaf wax is of microbial origin, rather than produced by the plant itself. Although the compound could only be detected in some *C. annuum* accessions, the tropane alkaloid does not seem to be related to thrips resistance (Table S2). Further studies are needed to determine the origin and the function of this alkaloid in *Capsicum*.

Some triterpenoids, the amyrins, were higher in resistant pepper accessions. Maharijaya et al. (2012) also found high concentrations of an unknown triterpenoid (phytosterol) in resistant pepper accessions. Triterpenoids are known to have a deterrent effect on feeding and oviposition of some insects, such as *Plutella xylostella* and *Phyllotreta nemorum* (Eigenbrode and Pillai 1998; Kuzina et al. 2009). Amyrins are the backbone structures for insect-deterrent saponins (Khakimov et al. 2015). In our study, the variation in α-amyrin levels among the different *C. annuum* accessions was considerable and therefore only weakly significant different between resistant and susceptible plants. β-Amyrin concentrations were low in the susceptible *C. chinense* accession compared to the resistant *C. chinense* accessions. However, within the *C. chinense* group our statistical power was low. Our analysis indicates that amyrins and structurally related compounds may serve as leads for thrips resistance breeding.

We tested thrips damage on the *Capsicum* accessions in three different thrips assays. Damage levels of the accessions varied among the three tests, but the least and most resistant accessions remained constant throughout all three trials (Visschers et al. 2019a, Macel et al. 2019). Plant resistance to insects is at least partly determined by plant age and by the environment, as is shown in many other studies (e.g. Damon et al. 2014; Visschers et al. [Bibr CR42][Bibr CR43]). Nevertheless, *Capsicum* accessions that had consistent relatively low feeding damage and thrips numbers, had low concentrations of the cuticular wax C_25_-C_29_ alkanes. Further validation with, for example, wax mutant lines could elucidate the role of these alkanes in thrips resistance or susceptibility. This could also reveal whether wax layer composition is more important for insect resistance than the total amount of wax (Aragon et al. 2017). The cuticular wax layer also plays an important role in resistance to pathogens and protection against desiccation and UV light. Breeding for thrips resistance in peppers by manipulating the leaf and fruit wax layer composition and quantity therefore will have to balance the different costs and benefits of these wax metabolites.

## Electronic Supplementary Material

ESM 1 (DOCX 29.8 KB)

ESM 2 (DOCX 218 KB)
