# Supplementary material for: High Concentrations of Very Long Chain Leaf Wax Alkanes of Thrips Susceptible Pepper Accessions (Capsicum spp)
Source: J Chem Ecol. 2020 Oct 22;46(11):1082–9. doi: 10.1007/s10886-020-01226-x (PMC7677282; doi:10.1007/s10886-020-01226-x)
Supplement: Supplementary file 1 — (DOCX 29.8 KB) [file 10886_2020_1226_MOESM1_ESM.docx]

**Supplementary Material** to Macel *et al*. “Cuticular leaf wax composition of thrips resistant and susceptible peppers (*Capsicum* spp.)”

**Table S1**. *Capsicum* source material from the Centre for Genetic Resources, the Netherlands (CGN); Radboud University (RU) codes, species and CGN numbers.

| RU code | *Capsicum* species | CGN number | Origin | Variety |
| --- | --- | --- | --- | --- |
| 63 | *C. annuum* | 23765 | Mexico | land variety |
| 23 | *C. annuum* | 22151 | China | land variety |
| 19 | *C. annuum* | 16913 | Germany | breeders variety |
| 34 | *C. annuum* | 23222 | USA | breeders variety |
| 43 | *C. annuum* | 17227 | Hungary | land variety |
| 52 | *C. annuum* | 21550 | Netherlands | breeders variety |
| 14 | *C. annuum* | 23289 | Zambia | land variety |
| 38 | *C. chinense* | 17004 | Suriname | land variety |
| 41 | *C. chinense* | 16994 | Brazil | land variety |
| 70 | *C. chinense* | 16995 | Brazil | land variety |
| 13 | *C. chinense* | 21557 | USA | research material |

**Table S2**. Mean concentrations (μg/dm^2^) of wax metabolites of *Capsicum* accessions. RU codes of accessions are indicated, RU#-S is accession classified as susceptible, RU#-R as resistant (*n*= 2-7 plants per accession). Rt = retention time.

|  |  |  | *Capsicum annuum* | | | | | | | *Capsicum chinense* | | | |
| --- | --- | --- | --- | --- | --- | --- | --- | --- | --- | --- | --- | --- | --- |
| **Compound** | **Formula** | **Rt** | **14-S** | **34-S** | **43-S** | **52-S** | **19-R** | **23-R** | **63-R** | **38-S** | **13-R** | **41-R** | **70-R** |
| Tropacocaine | C_15_H_19_NO_2_ | 9.73 | 0 | 0.05 | 0 | 0.07 | 0.02 | 0.61 | 0 | 0 | 0 | 0 | 0 |
| Pentacosane | C_25_H_52_ | 18.14 | 0.22 | 0.03 | 0.23 | 0.06 | 0.10 | 0 | 0 | 0 | 0 | 0 | 0 |
| Hexacosane | C_26_H_54_ | 20.19 | 0.55 | 0.22 | 0.26 | 0.95 | 0.05 | 0.02 | 0 | 0 | 0 | 0 | 0 |
| Heptacosane | C_27_H_56_ | 22.33 | 25.65 | 21.23 | 15.62 | 36.04 | 5.38 | 3.82 | 4.84 | 0.58 | 9.87 | 6.51 | 0.25 |
| Octacosane | C_28_H_58_ | 24.42 | 1.81 | 1.35 | 0.77 | 2.43 | 0.43 | 0.6 | 0.44 | 0.05 | 1.34 | 0.72 | 0.03 |
| Nonacosane | C_29_H_60_ | 26.53 | 11.76 | 9.02 | 8.14 | 12.59 | 6.12 | 5.56 | 3.43 | 2.52 | 8.13 | 6.57 | 2.19 |
| Triacotane | C_30_H_62_ | 28.58 | 0.75 | 0.58 | 0.66 | 0.44 | 0.60 | 0.53 | 0.44 | 0.57 | 0.41 | 0.29 | 0.18 |
| Hentriacotane | C_31_H_64_ | 30.65 | 16.61 | 14.67 | 26.22 | 7.72 | 20.57 | 13.65 | 11.73 | 11.36 | 9.71 | 14.27 | 11.48 |
| Dotriacotane | C_32_H_66_ | 32.62 | 2.05 | 1.98 | 3.62 | 0.44 | 4.01 | 2.25 | 2.77 | 1.29 | 0.69 | 1.82 | 2.05 |
| Tritriacotane | C_33_H_68_ | 34.61 | 22.94 | 22.32 | 52.92 | 4.84 | 39.68 | 14.64 | 21.81 | 11.76 | 6.75 | 12.15 | 13.13 |
| Tetratriacotane | C_34_H_70_ | 36.47 | 1.06 | 1.19 | 2.03 | 0.08 | 2.09 | 0.71 | 0.76 | 0.29 | 0.11 | 0.14 | 0.11 |
| Pentatriacotane | C_35_H_72_ | 38.35 | 6.94 | 7.82 | 22.55 | 2.05 | 16.22 | 2.35 | 2.46 | 2.85 | 2.39 | 1.61 | 0.33 |
| Heptatriacotane | C_37_H_76_ | 41.92 | 1.27 | 0.80 | 2.95 | 0.30 | 1.83 | 0.20 | 0 | 0.32 | 0.57 | 0.02 | 0 |
| *iso*-nonacosane | C_29_H_60_ | 25.72 | 0.88 | 0.81 | 1.14 | 2.29 | 0.21 | 0.07 | 0.02 | 0 | 0 | 0 | 0 |
| *iso*-hentriacotane | C_31_H_64_ | 29.86 | 1.82 | 1.93 | 2.29 | 2.50 | 1.21 | 1.62 | 0.27 | 0.29 | 0.56 | 0.98 | 0.29 |
| branched alkane | C_32_H_66_ | 32.11 | 0.39 | 0.27 | 0.64 | 0.77 | 0.48 | 0.86 | 0.02 | 0.27 | 0.32 | 0.19 | 0.30 |
| *iso*-tritiacotane | C_33_H_68_ | 33.86 | 4.26 | 4.24 | 8.00 | 3.57 | 4.56 | 3.63 | 1.79 | 1.92 | 0.98 | 5.17 | 3.37 |
| branched alkane |  | 36.77 | 0 | 0.04 | 0.21 | 0.48 | 0.04 | 0.11 | 0 | 0.08 | 0.16 | 0 | 0.05 |
| *iso*-pentatriacotane | C_35_H_72_ | 37.64 | 1.68 | 1.07 | 2.43 | 1.35 | 1.10 | 0.60 | 0.21 | 0.24 | 0.05 | 0.51 | 0.11 |
| *iso-*hexatriacotane | C_36_H_74_ | 39.70 | 0.39 | 0.06 | 0.21 | 0.57 | 0.04 | 0.04 | 0 | 0.19 | 0 | 0.03 | 0.32 |
| *iso*-heptiatriacotane | C_37_H_76_ | 41.25 | 0.31 | 0.11 | 0.18 | 0.25 | 0.03 | 0.01 | 0 | 0.02 | 0 | 0 | 0 |
| 1-hexacosanol | C_26_H_54_O | 27.58 | 0.88 | 0.63 | 2.48 | 0.56 | 1.50 | 0.21 | 0 | 0 | 0 | 0 | 0 |
| 1-heptacosanol | C_27_H_56_O | 29.62 | 0.36 | 0.18 | 0.59 | 0.01 | 0.31 | 0.08 | 0 | 0 | 0 | 0 | 0 |
| 1-octacosanol | C_28_H_58_O | 31.70 | 100.38 | 55.11 | 108.46 | 29.85 | 144.90 | 35.71 | 14.94 | 30.47 | 3.41 | 21.05 | 12.00 |
| 1-nonacosanol | C_29_H_60_O | 33.57 | 6.07 | 5.38 | 7.72 | 1.13 | 9.14 | 2.86 | 0.78 | 4.44 | 0.93 | 5.18 | 3.18 |
| 1-triacotanol | C_30_H_62_O | 35.53 | 39.90 | 33.41 | 62.99 | 10.78 | 56.41 | 21.92 | 7.17 | 82.19 | 19.06 | 117.89 | 77.01 |
| 1-henatriacotanol | C_31_H_64_O | 37.31 | 0.24 | 0.25 | 0.68 | 0 | 0.64 | 0.09 | 0.03 | 0.74 | 0.14 | 1.33 | 2.12 |
| 1-dotriacontanol | C_32_H_66_O | 39.15 | 6.81 | 0.02 | 1.25 | 0.02 | 0.89 | 0.49 | 0.01 | 9.06 | 2.07 | 11.68 | 15.52 |
| alkyl ester |  | 32.92 | 0 | 0.31 | 1.14 | 2.32 | 0.26 | 0 | 0 | 0.03 | 0 | 0 | 0 |
| ß-sitosterol | C_29_H_50_O | 35.33 | 6.90 | 12.72 | 8.89 | 5.52 | 13.81 | 5.83 | 3.40 | 0.48 | 2.03 | 1.65 | 3.05 |
| Triterpenoid |  | 35.69 | 1.85 | 3.72 | 4.87 | 0.66 | 6.10 | 0.04 | 0.82 | 0 | 0 | 0 | 0.01 |
| ß-amyrin | C_30_H_50_O | 36.0 | 1.22 | 3.47 | 2.03 | 3.49 | 3.21 | 9.24 | 3.10 | 0.20 | 3.13 | 3.09 | 3.72 |
| α-amyrin | C_30_H_50_O | 36.19 | 1.35 | 0.97 | 0.80 | 1.57 | 0.40 | 5.37 | 1.21 | 1.61 | 0.85 | 3.07 | 1.23 |
| Triterpenoid |  | 37.88 | 0.50 | 0.08 | 0.33 | 0.02 | 0.26 | 1.02 | 0.14 | 0.03 | 0 | 0 | 0 |
| **Total Wax** |  |  | **269.67** | **234.59** | **389.96** | **136.56** | **352.16** | **135.76** | **82.61** | **164.38** | **74.77** | **215.92** | **152.40** |
