# Supplementary material for: High Concentrations of Very Long Chain Leaf Wax Alkanes of Thrips Susceptible Pepper Accessions (Capsicum spp)
Source: J Chem Ecol. 2020 Oct 22;46(11):1082–9. doi: 10.1007/s10886-020-01226-x (PMC7677282; doi:10.1007/s10886-020-01226-x)

**Figure S1**. **A)** Example of a GC-MS chromatogram of the leaf wax metabolites of *Capsicum annuum* and **B)** the mass spectrum of the tropane alkaloid peak at 9.73 minutes.


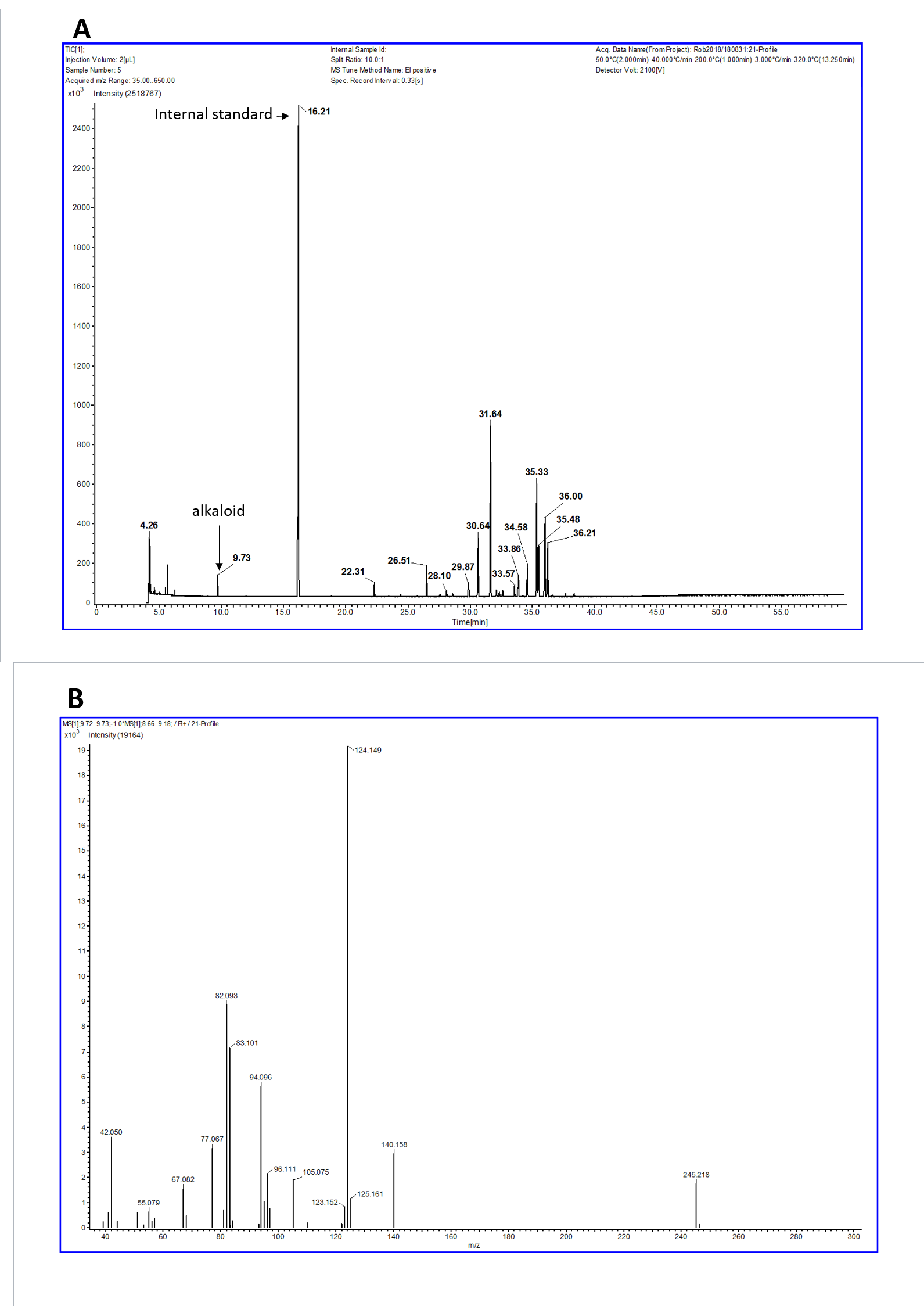

Supplement: Supplementary file 2 — (DOCX 218 KB) [file 10886_2020_1226_MOESM2_ESM.docx]
